# Concentration- and schedule-dependent effects of chemotherapy on the angiogenic potential and drug sensitivity of vascular endothelial cells

**DOI:** 10.1007/s10456-012-9321-x

**Published:** 2012-11-10

**Authors:** Eddy Pasquier, Maria-Pia Tuset, Janine Street, Snega Sinnappan, Karen L. MacKenzie, Diane Braguer, Nicolas Andre, Maria Kavallaris

**Affiliations:** 1 Children’s Cancer Institute Australia for Medical Research, Lowy Cancer Research Centre, UNSW, PO Box 81, Randwick, NSW 2031 Australia; 2 Metronomics Global Health Initiative, Marseille, France; 3 Faculty of Pharmacy, INSERM UMR 911, Centre de Recherche en Oncologie biologique et Oncopharmacologie, Aix-Marseille University, Marseille, France; 4 Hematology and Pediatric Oncology Department, La Timone University Hospital of Marseille, Marseille, France; 5 Australian Centre for Nanomedicine, University of New South Wales, Kensington, NSW 2051 Australia; 6 Present Address: Kolling Institute of Medical Research, Royal North Shore Hospital, St Leonards, NSW 2065 Australia

**Keywords:** Cancer, Angiogenesis, Metronomic chemotherapy, Vascular endothelial cells, ABC transporters, βIII-tubulin

## Abstract

The anti-angiogenic activity of chemotherapy is both dose- and schedule-dependent. While conventional maximum tolerated dose (MTD) chemotherapy exerts only mild and reversible anti-angiogenic effects, low-dose metronomic (LDM) chemotherapy was developed to specifically target tumour angiogenesis. However, the long-term effects of either MTD or LDM chemotherapy on vascular endothelial cells have never been investigated. Here, we demonstrated that repeated exposure to MTD and LDM chemotherapy differentially impact on the angiogenic potential and chemosensitivity of immortalized endothelial cells. Repeated MTD vinblastine treatment of vascular endothelial cells led to an increased proliferation rate and resistance to paclitaxel. In contrast, repeated LDM treatment with vinblastine or etoposide impaired the angiogenic potential of endothelial cells and increased their chemosensitivity. This effect was associated with a significant decrease in βII- and βIII-tubulin expression. Functional analysis using siRNA showed that silencing the expression of βIII-tubulin in endothelial cells significantly decreased their capacity to form vascular structures and increased their sensitivity to the anti-angiogenic and vascular-disrupting effects of chemotherapy, whereas silencing βII-tubulin expression had no effect. Collectively our results show that LDM chemotherapy impairs the angiogenic potential of endothelial cells while increasing their chemosensitivity—an effect at least in part mediated by the down-regulation of βIII-tubulin expression. Furthermore, our study suggests that βIII-tubulin represents an attractive therapeutic target to increase the anti-angiogenic effects of chemotherapy and overall anti-tumour efficacy.

**Electronic supplementary material:**

The online version of this article (doi:10.1007/s10456-012-9321-x) contains supplementary material, which is available to authorized users.

## Introduction

The discovery of the central role played by tumour angiogenesis in cancer progression and metastasis has not only led to the development of novel anti-cancer drugs specifically targeting this key biological process but also instigated the re-examination of conventional chemotherapy agents. A number of studies thus revealed that many of the chemotherapeutic drugs commonly used in the clinic are effective at treating cancer because they can affect both the cancer cells and the tumour vasculature [1–3]. This led to the development of novel treatment modalities aimed at promoting the anti-angiogenic activity of chemotherapy. These new treatment schedules, called metronomic chemotherapy or metronomic scheduling of anti-cancer treatment (MSAT) [4–6], are based on more frequent administration of chemotherapeutic drugs at relatively low dose, as compared to conventional chemotherapy, and with no prolonged drug-free interval. Following initial demonstration of efficacy in pre-clinical models [1, 7], metronomic chemotherapy has shown promising results in a number of clinical applications (reviewed in [5, 6]), such as advanced breast cancer in adults [8, 9] or recurrent medulloblastoma in children [10]. Furthermore, the efficacy of metronomic chemotherapy is currently investigated in several phase II and III clinical trials worldwide in a variety of human malignancies (http//:clinicaltrials.gov).

Despite accumulating evidence of clinical benefits, the complex and multi-faceted mechanism of action of metronomic chemotherapy has only been partially unveiled and warrants further investigation. One of the initial rationales for the development of anti-angiogenic therapies in general, and metronomic chemotherapy in particular, was based on the idea that, unlike cancer cells, vascular endothelial cells are genetically stable and thus less likely to acquire drug resistance [1, 11, 12]. However, accumulating evidence suggests that this hypothesis is, at least in part, incorrect. Endothelial cells isolated from tumour blood vessels were found to display tumour-specific genetic abnormalities [13–15]. In addition, studies have shown that tumour endothelial cells can be intrinsically resistant to chemotherapeutic drugs, such as adriamycin and temozolomide [16, 17], and even acquire drug resistance through VEGF-induced up-regulation of MDR1 expression [18]. The long-term effects of chemotherapy on vascular endothelial cells remains however unexplored.

Recent studies revealed that chemotherapy dosing schedule strongly influences drug resistance development in cancer cells. Weekly docetaxel treatment was found to induce the up-regulation of a number of genes involved in drug resistance, including *TUBB3* (i.e. the gene encoding β-III tubulin) and ATP-binding cassette (ABC) transporters *ABCB1* and *ABCC10*, in xenografted ovarian tumours whereas continuous docetaxel treatment did not [19]. Furthermore, Kerbel and colleagues demonstrated that extended low-dose metronomic (LDM) cyclophosphamide therapy could lead to drug resistance in prostate cancer cells in vivo but these cells retained sensitivity to maximum-tolerated dose (MTD) cyclophosphamide [20]. This strongly suggests that resistance to LDM chemotherapy in cancer cells imparts distinct mechanisms of resistance to MTD chemotherapy. Here, we hypothesized that, similarly to what was reported in tumour cells, repeated exposure to either LDM or MTD chemotherapy would differentially impact on the angiogenic potential and chemosensitivity of endothelial cells, through modulation of the expression of genes involved in drug resistance, such as ABC transporters and β-tubulin isotypes.

## Materials and methods

### Cell culture

BMH29L cells are bone marrow-derived endothelial cells (BMECs) that were immortalized by ectopic expression of human telomerase reverse transcriptase [21]. They were grown in Medium 199 (Invitrogen) containing 10 % heat-inactivated Fetal Calf Serum (FCS), 5 % male human serum, AB only (Sigma-Aldrich, Castle Hill, Australia), 1 % penicillin and streptomycin, 1 % heparin, 5 ng/mL recombinant human FGF_β_ (fibroblast growth factor β; Sigma-Aldrich) and 20 μg/mL Endothelial Cell Growth Factor (Roche, Dee Why, Australia). HMEC-1 (Human Microvascular Endothelial Cell line 1) cells were originally developed by Prof Ades [22] and obtained from the Cell Culture Laboratory in the Hôpital de la Conception (Assistance Publique Hôpitaux de Marseille, Marseille, France). They were grown in MCDB-131 medium (Invitrogen, Mount Waverley, Australia) containing 10 % heat-inactivated FCS, 2 mM l-glutamine, 1 % penicillin and streptomycin, 1 μg/mL hydrocortisone and 10 ng/mL epithelial growth factor (BioScientific, Gymea, Australia). Both cell lines were routinely maintained in culture on 0.1 % gelatin-coated flasks at 37 °C and 5 % CO_2_. Cell lines were regularly screened and are free from mycoplasma contamination.

### Long-term drug treatment

BMH29L cells were incubated with vinblastine (*VLB*) or etoposide (*VP16*) following a maximum-tolerated dose (*MTD*) conventional schedule or a low-dose metronomic (*LDM*) schedule for a total duration of 100 days (Online Resource 1). In the MTD schedule, BMH29L cells were treated every 2 weeks for a total of 7 courses with drug-free medium (*Ctrl*-*MTD*) or containing VLB or VP16 at the IC_80_ for cell proliferation (i.e. 20 nM and 50 μM of VLB and VP16, respectively). In the LDM schedule, BMH29L cells were treated 5 days a week for a total of 14 courses with drug-free medium (*Ctrl*-*LDM*) or containing VLB or VP16 at the highest non-toxic concentration (i.e. 1 nM and 0.5 μM of VLB and VP16, respectively). This led to the establishment of 6 new BMH29L subclones: *Ctrl*-*LDM*, *Ctrl*-*MTD*, *VLB*-*LDM*, *VLB*-*MTD*, *VP16*-*LDM* and *VP16*-*MTD*. These subclones were cryopreserved at the end of the long-term treatment and not kept in culture for more than 1 month for all subsequent experiments.

### Doubling time and growth inhibition assay

Following long-term drug treatment, BMH29L subclones were seeded onto 6-well plates at a cell density of 20,000 cells/well and counted every 24 h for 4 days using the trypan blue exclusion method. Doubling times were determined by mathematical regression using GraphPad Prism 4 software (GraphPad Software Inc, La Jolla, CA). Growth inhibition assays were performed as previously described [23]. Briefly, cells were seeded at 1,500 cells/well (BMH29L) or 3,750 cells/well (HMEC-1) in 96-well gelatin-coated plates. After 24 h, cells were treated with a range of concentrations of chemotherapeutic drugs and after 72 h drug incubation, metabolic activity was detected by addition of Alamar blue and spectrophotometric analysis. Cell proliferation was determined and expressed as a percentage of untreated control cells. The determination of IC_50_ and IC_80_ values was performed by point-to-point fit spline analysis using GraphPad Prism 4 software.

### Quantitative RT-PCR

The expression of ABC transporters *ABCB1*, *ABCC1*, *ABCC2* and *ABCC10* and β-tubulin genes *TUBB*, *TUBB2A* and *TUBB3* was examined in BMH29L subclones using real-time quantitative RT-PCR, as previously described [24, 25]. Total RNA was extracted and DNAse treated using the Qiagen Mini RNeasy kit according to the manufacturer instructions (Qiagen, Doncaster, Australia). cDNA synthesis was performed using High capacity cDNA reverse transcription kit with RNAse inhibitor according to the manufacturer instructions (Applied Biosystem, Melbourne, Australia). Real time PCR was run on 7900HT Fast Real-Time PCR system using either Taqman^®^ gene expression assays (Applied Biosystems) for *ABCB1* (Hs00184500), *ABCC1* (Hs01561503), *ABCC10* (Hs00375716) and endogenous control *HPRT1* (4326321E) or Power SYBR^®^ green (Applied Biosystems) for *TUBB* (QT00089775), *TUBB2A* (QT01677326), *TUBB3* (QT00083713) and endogenous control *GAPDH* (QT01192646). *ABCC2* forward and reverse primer sequences were 5′-AGAGAACAGCTTTCGTCGAACAC-3′ and 5′-CATTCCGAGTTTTCAAGGAGTTTC-3′, respectively. *ABCC2* probe sequence was ACCTAGAACTGCGGCTA. Gene expression levels were determined using the ΔΔ*C*_t_ method, normalized to the *HPRT1* control for ABC transporters and the *GAPDH* control for β-tubulin genes, and expressed relative to a calibrator [26].

### Radiolabelled drug accumulation assay

For drug accumulation studies, BMH29L subclones, seeded in 12-well plates, were incubated for 4 h at 37 °C with [^3^H]-vincristine (15.8 Ci/mmol; final concentration 50 nM) in presence or absence of 10 μM verapamil. Cells were then washed thrice with ice-cold PBS to eliminate the extracellular tritiated drug and lysed in 0.5 M NaOH. Intracellular [^3^H]-vincristine concentration was determined by β-scintillation counting and normalized to protein content, as determined by BCA assay [27].

### In vitro Matrigel™ assay

Matrigel™ (BD Biosciences, North Ryde, Australia) assay was used to determine the effects of repeated exposure to chemotherapy and βII and βIII tubulin knockdown on the angiogenic potential and chemosensitivity of endothelial cells, as previously described [23]. For the anti-angiogenesis analysis, cells were treated with different drug solutions 20 min after seeding on Matrigel and photographs were taken after 8 h drug incubation using the 5X objective of an Axiovert 200 M fluorescent microscope coupled to an AxioCamMR3 camera driven by the AxioVision 4.7 software (Carl Zeiss, North Ryde, Australia). For the vascular-disruption analysis, cells were allowed to undergo morphogenesis and form capillary-like structures for 6 h before drug treatment was initiated. Photographs were then taken using the same microscope device after 2 h drug incubation. The anti-angiogenic and vascular-disrupting effects were then quantitatively evaluated by measuring the total surface area of capillary tubes formed in at least 10 view fields per well using AxioVision 4.7 software.

### Gene silencing by small interfering RNA

βII- and βIII-tubulin gene expression were silenced in endothelial cells using siRNA sequences whose potency and specificity have been validated previously [28, 29] and obtained from Dharmacon (Thermo Fisher Scientific, Scoresby, Australia) and Qiagen (Qiagen), respectively. The optimum amount of siRNA was determined to be 200 pmol (data not shown) and was used in all subsequent experiments. A non-silencing control siRNA, which has no sequence homology to any known human gene sequence, was used as a negative control in all experiments (Qiagen). Cells were transfected using the Nucleofector^®^ II device (Lonza, Mount Waverley, Australia) as previously described [30]. Briefly, HMEC-1 and BMH29L cells were resuspended in nucleofector^®^ solution R and V, respectively, and transfected with siRNA using specific nucleofector^®^ programs (T-016 and S-003 for HMEC-1 and BMH29L, respectively). In all subsequent experiments, drug treatment was initiated 72 h after siRNA transfection—when the knockdown of the targeted gene was the most effective.

### Western blotting analysis

Total cellular proteins from endothelial cells (10–15 μg) were resolved on 12 % SDS-PAGE before electrotransfer onto nitrocellulose membrane. Immunoblotting was done using antibodies directed against βI-tubulin (clone SAP 4G5; Abcam), βII-tubulin (clone 7B9; Chemicon), βIII-tubulin (clone TUJ1; Chemicon), βIV-tubulin (clone ONS 1A6; Sigma-Aldrich), total β-tubulin (clone TUB 2.1; Sigma-Aldrich) and glyceraldehyde-3-phosphate dehydrogenase (GADPH; Abcam). The membranes were then incubated with horseradish peroxidase–conjugated IgG secondary antibodies, and protein was detected with ECL Plus (GE Healthcare Life Sciences). The blots were scanned and densitometric analysis performed as previously described [31].

### Statistical analysis

All experiments were performed at least in triplicate. Statistical significance was determined using two-sided student’s t-test in the GraphPad Prism 4 software (GraphPad Software, Inc).

## Results

### Conventional and metronomic chemotherapy differentially impact on the angiogenic potential of vascular endothelial cells

**Fig. 1:**
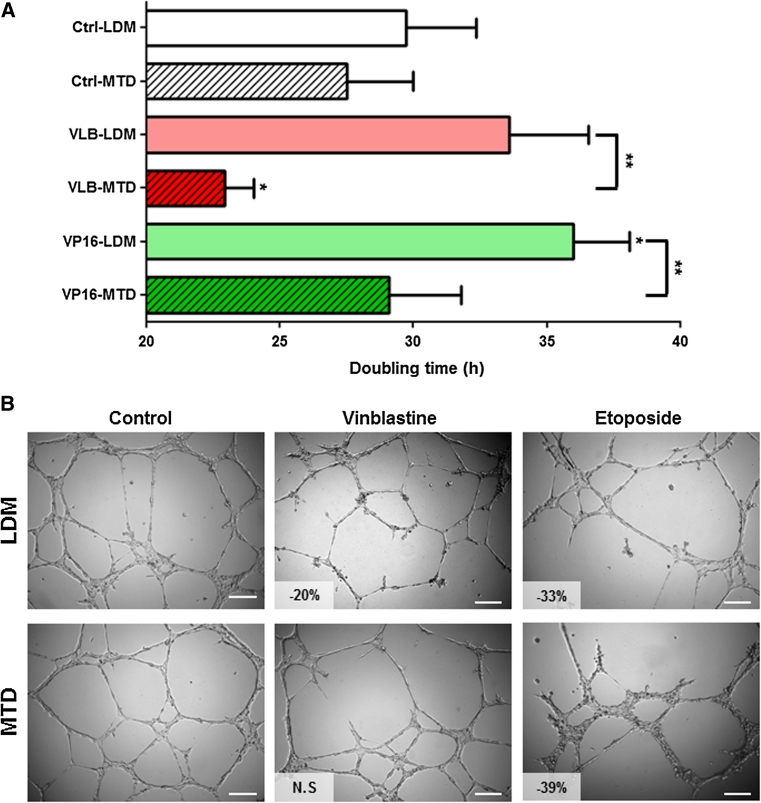
Impact of repeated exposure to chemotherapy on the angiogenic potential of endothelial cells. **a** Doubling time of the 6 endothelial subclones established by incubating BMH29L endothelial cells for 100 days with no drug (*Ctrl*-*LDM* and *Ctrl*-*MTD*) or with vinblastine (*VLB*) or etoposide (*VP16*) following a low-dose metronomic (*LDM*) schedule (i.e. treatment 5 days/week with highest non-toxic concentration) or a maximum tolerated dose (*MTD*) schedule (i.e. treatment every 2 weeks with IC_80_). *Columns* means of four individual experiments, *bars* SE. Statistics were calculated by comparing drug-treated cells with control untreated cells, unless indicated otherwise; *p < 0.05; **p < 0.01. **b** Representative photographs of the 6 BMH29L subclones following 8 h incubation on Matrigel. Vascular structures were imaged on a Zeiss Axiovert 200 M using a 5X objective. Percentage of angiogenesis inhibition as compared to control cells is indicated; *NS* non-significant, *Scale bar* 250 μm

Bone marrow-derived endothelial cells immortalized by ectopic expression of human telomerase reverse transcriptase [21] were treated for 100 days with vinblastine (*VLB*) or etoposide (*VP16*) following a low-dose metronomic (*LDM*) or a maximum-tolerated dose (*MTD*) schedule (Online Resource 1). This led to the establishment of 6 BMH29L subclones: *Ctrl*-*LDM*, *Ctrl*-*MTD*, *VLB*-*LDM*, *VLB*-*MTD*, *VP16*-*LDM* and *VP16*-*MTD*. The impact of repeated exposure to chemotherapy on the doubling time of these subclones was determined by proliferation assay (Fig. 1a). BMH29L cells treated fortnightly with vinblastine at high concentration (*VLB*-*MTD*) proliferated at a faster rate as compared to control cells (22.9 ± 1.1 h and 27.5 ± 2.5 h for *VLB*-*MTD* and *Ctrl*-*MTD* cells, respectively; *p* < 0.05). In contrast, BMH29L cells treated 5 days a week with etoposide at low concentration (*VP16*-*LDM*) had a significantly slower doubling time as compared to control cells (36.0 ± 2.1 h and 29.7 ± 2.6 h for *VP16*-*LDM* and *Ctrl*-*LDM* cells, respectively; *p* < 0.05). Furthermore, repeated exposure to LDM chemotherapy resulted in a significantly slower doubling time of endothelial cells as compared to MTD chemotherapy (*p* < 0.01).

Matrigel assay was then used to analyse the effects of long-term chemotherapy treatment on the capacity of endothelial cells to undergo morphological differentiation into vascular structures in vitro (Fig. 1b). Repeated exposure to etoposide resulted in impaired angiogenic properties, irrespective of the treatment schedule (−33 and −39 % vessel formation for *VP16*-*LDM* and *VP16*-*MTD* cells as compared to control cells, respectively; *p* < 0.05). In contrast, the impact of vinblastine on the angiogenic potential of endothelial cells was schedule-dependent. *VLB*-*LDM* cells displayed impaired angiogenic properties (−20 % as compared to control cells; *p* < 0.05) whereas *VLB*-*MTD* cells were able to form vascular structures to the same extent as control untreated cells.

### Conventional and metronomic chemotherapy differentially impact on the chemosensitivity of vascular endothelial cells

**Fig. 2:**
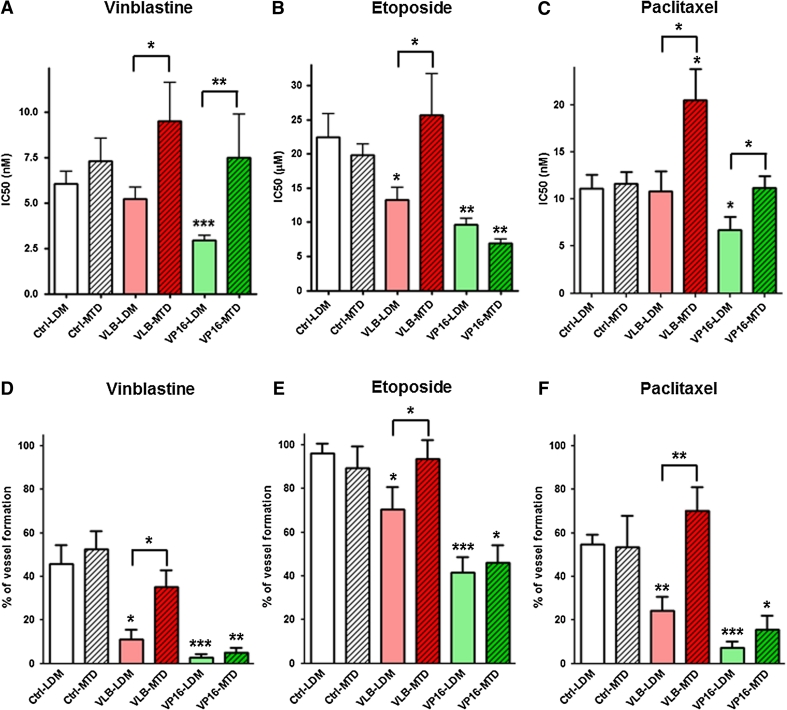
Impact of repeated exposure to chemotherapy on the chemosensitivity of endothelial cells. **a**–**c** Histograms showing the IC_50_ values of vinblastine (**a**), etoposide (**b**) and paclitaxel (**c**) in the 6 BMH29L subclones as determined by 72 h growth inhibition assay. **d**–**f** Percentage of vascular structure formation following 8 h incubation on Matrigel with 5 nM vinblastine (**d**), 10 μM etoposide (**e**) and 10 nM paclitaxel (**f**). *Columns* means of at least four individual experiments, *bars* SE. Statistics were calculated by comparing drug-treated cells with control cells, unless indicated otherwise; *p < 0.05; **p < 0.01; ***p < 0.001

Short-term growth inhibition assay with vinblastine, etoposide and paclitaxel (PTX) was used to determine whether repeated exposure to chemotherapy could result in acquisition of drug resistance in vascular endothelial cells. We found that the long-term effects of chemotherapy on the chemosensitivity of endothelial cells were schedule-dependent (Fig. 2). While repeated exposure to vinblastine at MTD increased the resistance of BMH29L to paclitaxel by 76 % as compared to control untreated cells (Fig. 2c; *p* < 0.05), repeated exposure to LDM chemotherapy did not induce resistance but rather increased the chemosensitivity of endothelial cells. *VLB*-*LDM* cells were found to have a 41 % increase in sensitivity to etoposide (Fig. 2b; *p* < 0.05) and *VP16*-*LDM* cells a 51, 57 and 40 % increase in sensitivity to vinblastine (Fig. 2a; *p* < 0.001), etoposide (Fig. 2b; *p* < 0.01) and paclitaxel (Fig. 2c; *p* < 0.05) as compared to control cells, respectively. In addition, except for repeated exposure to etoposide, which resulted in increased sensitivity to etoposide irrespective of the treatment schedule, long-term treatment with LDM chemotherapy increased the overall drug sensitivity of endothelial cells as compared to cells exposed to MTD chemotherapy (Fig. 2a–c; *p* < 0.05).

Angiogenesis assay further confirmed these results. Repeated exposure to etoposide resulted in increased sensitivity to the anti-angiogenic effects of vinblastine (Fig. 2d; *p* < 0.01), etoposide (Fig. 2e; *p* < 0.05) and paclitaxel (Fig. 2f; *p* < 0.05), irrespective of the treatment schedule. Long-term effects of vinblastine however were schedule-dependent. Repeated exposure to vinblastine at MTD did not affect the sensitivity of endothelial cells to the anti-angiogenic activity of chemotherapeutic drugs whereas repeated exposure to vinblastine at low concentration significantly increased sensitivity (Fig. 2d–f; *p* < 0.05).

### Repeated exposure to chemotherapy modulates the expression of ABC transporter without dramatically affecting intracellular drug accumulation

**Fig. 3:**
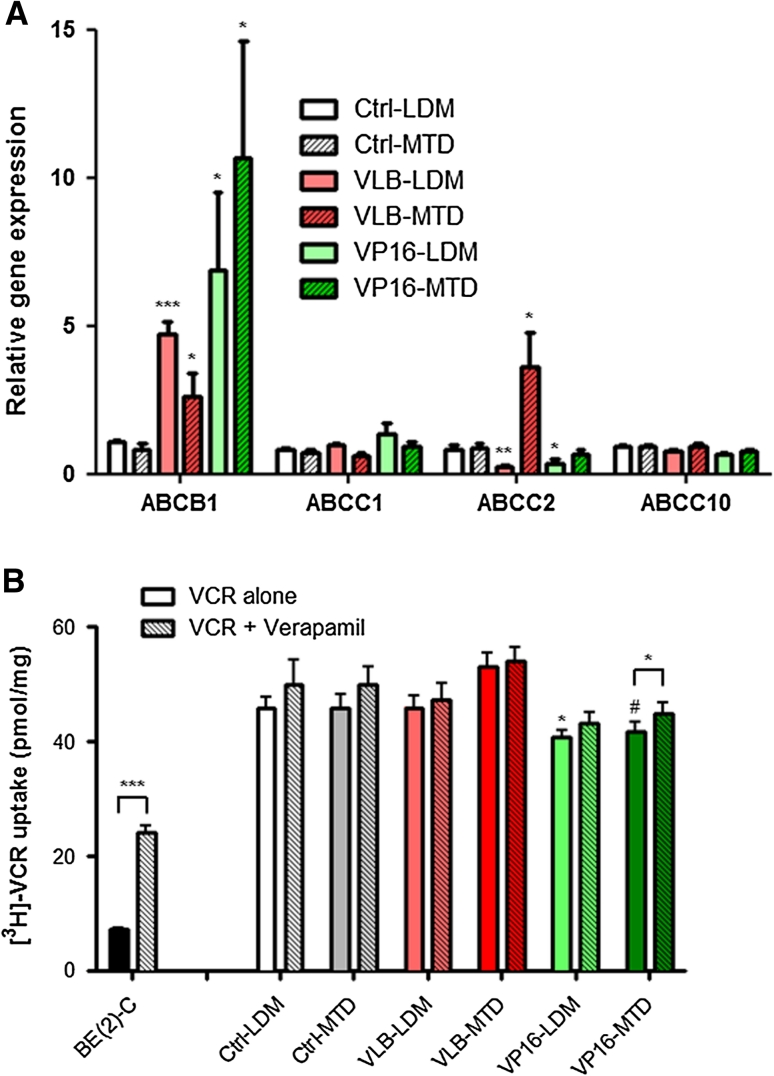
Impact of repeated exposure to chemotherapy on ABC transporter expression and intracellular drug accumulation. **a** Relative gene expression of ABC transporters as determined by qRT-PCR using *HPRT1* as control gene. **b** Accumulation of [^3^H]-VCR after 4 h incubation in presence or absence of 10 μM verapamil. The BE(2)-C neuroblastoma cell line expressing high levels of P-gp was included as a positive control. *Columns* means of four individual experiments, *bars* SE. Statistics were calculated by comparing drug-treated cells with control cells, unless indicated otherwise; ^#^ *p* = 0.06; *p < 0.05; **p < 0.01; ***p < 0.001

To gain insights into the mechanisms involved in the differential chemosensitivity of endothelial cells following repeated exposure to chemotherapy, the gene expression level of ATP-binding cassette (ABC) transporters was assessed using quantitative real-time RT-PCR. Our results showed that long-term treatment with chemotherapy resulted in a significant increase (2.6- to 10.6-fold increase as compared to control untreated cells; *p* < 0.05) in *ABCB1* expression—the gene encoding the P-glycoprotein (P-gp)—irrespective of the treatment schedule (Fig. 3a). The increase in P-gp expression was confirmed at the protein level by immunoblotting (data not shown). In contrast, the effect of chemotherapy on *ABCC2* expression was schedule-dependent. *VLB*-*MTD* cells showed a significant increase in *ABCC2* expression (4.1-fold as compared to *Ctrl*-*MTD* cells; *p* < 0.05) whereas both subclones treated with LDM chemotherapy, *VLB*-*LDM* and *VP16*-*LDM*, displayed decreased levels of *ABCC2* (−73 and −62 % as compared to control cells, respectively; *p* < 0.05). No significant change in the expression of *ABCC1* and *ABCC10* was observed across the 6 BMH29L subclones.

Radiolabelled drug uptake studies were then performed to determine whether the changes in *ABCB1* and *ABCC2* gene expression were functional and associated with altered intracellular accumulation of the P-gp and ABCC2 substrate, vincristine. Our results showed that repeated exposure to chemotherapy only marginally altered the intracellular accumulation of tritiated vincristine ([^3^H]-VCR) in endothelial cells (Fig. 3b). Repeated treatment with etoposide thus led to a slight decrease in [^3^H]-VCR uptake (−11 and −9 % for *VP16*-*LDM* and *VP16*-*MTD* as compared to control cells; *p* < 0.06) whereas repeated exposure to vinblastine did not. Furthermore, the addition of P-gp inhibitor, verapamil, did not significantly increase [^3^H]-VCR uptake in the BMH29L subclones, with the exception of a 7 % increase observed in *VP16*-*MTD* cells (*p* < 0.05). This is in marked contrast with the 234 % increase in [^3^H]-VCR uptake induced by verapamil in P-gp expressing neuroblastoma cells [32]. Our results thus demonstrated that the increase in *ABCB1* gene expression observed following repeated exposure to chemotherapy did not translate into an increase in P-gp activity.

### Repeated exposure to chemotherapy induces significant changes in β-tubulin isotype expression

**Fig. 4:**
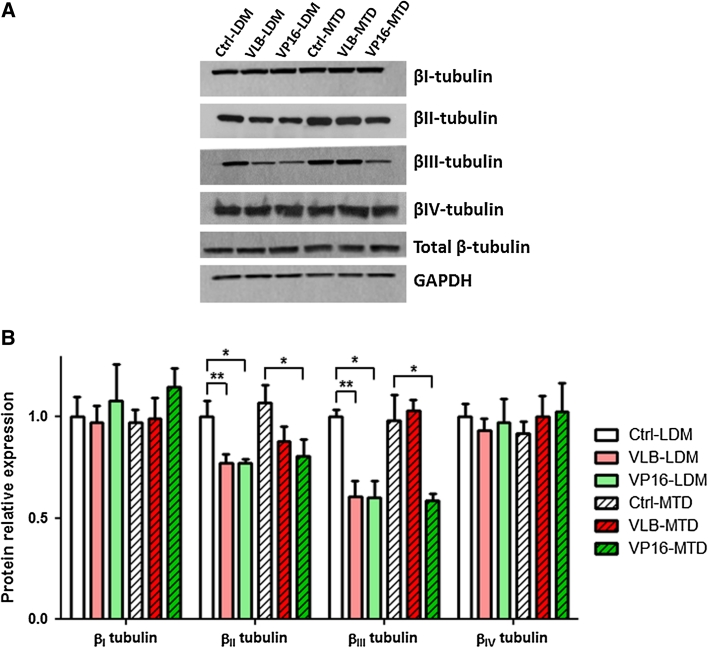
Effect of repeated exposure to chemotherapy on the expression of β-tubulin isotypes in endothelial cells. **a** Representative immunoblots of total endothelial cell lysates following long-term treatment with chemotherapy. Membranes were probed with antibodies directed against GADPH (loading control), βI-, βII-, βIII-, βIV- and total β-tubulin. **b** Histogram showing the relative expression of tubulin isotypes in the 6 BMH29L subclones as determined by densitometry after normalization with GADPH. *Columns* means of three individual experiments, *bars* SE. Statistics were calculated by comparing drug-treated cells with control cells; *p < 0.05; **p < 0.01

Our previous work and that of others have shown that changes in the expression level of specific β-tubulin isotypes can modulate the chemosensitivity of cancer cells not only to microtubule-targeting agents but also to other classes of chemotherapeutic drugs, such as DNA-damaging agents (reviewed in [33]). In order to gain better insights into how repeated exposure to chemotherapy affect the angiogenic potential and chemosensitivity of endothelial cells, the expression level of β-tubulin isotypes was assessed in the 6 BMH29L subclones by western blotting (Fig. 4). No significant difference in protein expression of βI-, βIV- and total β-tubulin was observed across the 6 subclones. However, a 40 ± 1 % decrease in βIII-tubulin protein expression was observed in *VLB*-*LDM*, *VP16*-*LDM* and *VP16*-*MTD* cells as compared to control cells (*p* < 0.05). In addition, these cells also displayed a 21 ± 2 % decrease in βII-tubulin protein expression as compared to control cells (*p* < 0.05). The decrease in βII and βIII-tubulin protein expression was associated with a significant decrease in *TUBB2A* and *TUBB3* (i.e. the genes encoding βII- and βIII-tubulin, respectively) mRNA levels in *VLB*-*LDM* and *VP16*-*MTD* cells (Online Resource 2), suggesting that the repression occurred at the transcriptional level. Collectively, these results show that repeated exposure to chemotherapy, especially when administered following a LDM schedule, resulted in the down-regulation of specific tubulin isotypes in vascular endothelial cells.

### βIII-tubulin expression mediates the sensitivity of endothelial cells to the anti-angiogenic effects of chemotherapy

**Fig. 5:**
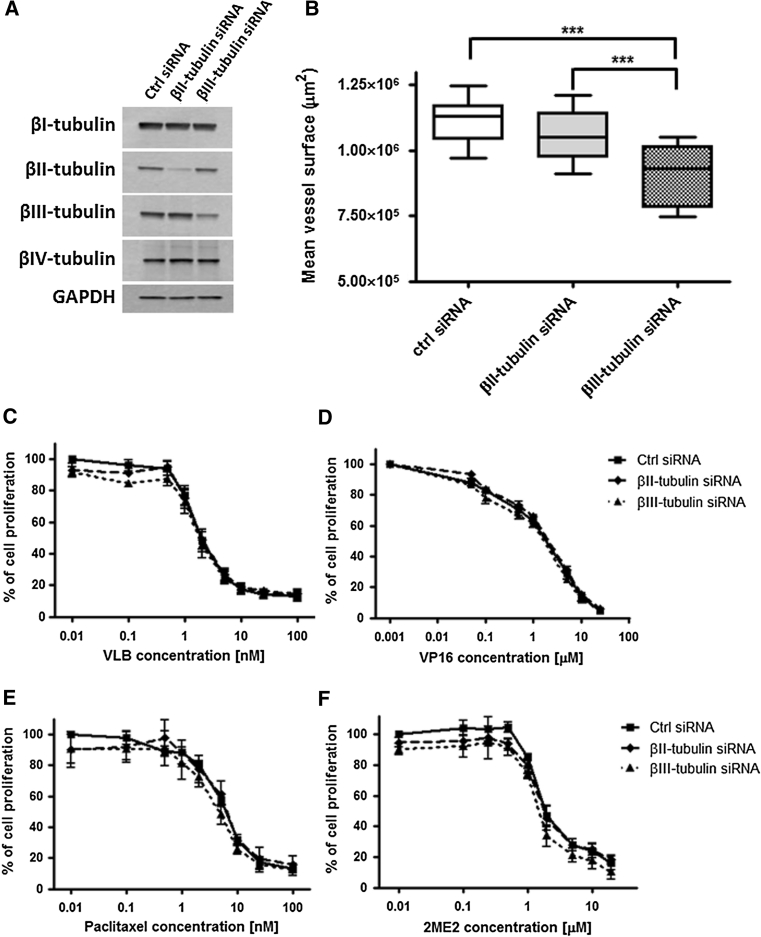
Functional study of βII- and βIII-tubulin in endothelial cells. **a** Representative immunoblots of whole cell lysates, 72 h after transfection of HMEC-1 cells with negative control, βII- and βIII-tubulin siRNA. Membranes were probed with antibodies directed against GADPH (loading control), βI-, βII-, βIII- and βIV-tubulin. **b** Mean surface occupied by vascular structures formed by HMEC-1 cells 72 h after siRNA transfection and following 8 h incubation on Matrigel. *Boxes* min–max range of at least 6 individual experiments, *bars* SD; ***p < 0.001. **c**–**f** Growth inhibition assays performed on siRNA-transfected HMEC-1 cells using Alamar Blue after 72 h incubation with a range of concentrations of vinblastine (**c**), etoposide (**d**), paclitaxel (**e**) and 2-methoxyestradiol (**f**). *Points* % of cell proliferation as compared to untreated control cells, means of at least three individual experiments, *bars* SE; log scale for x axis

**Fig. 6:**
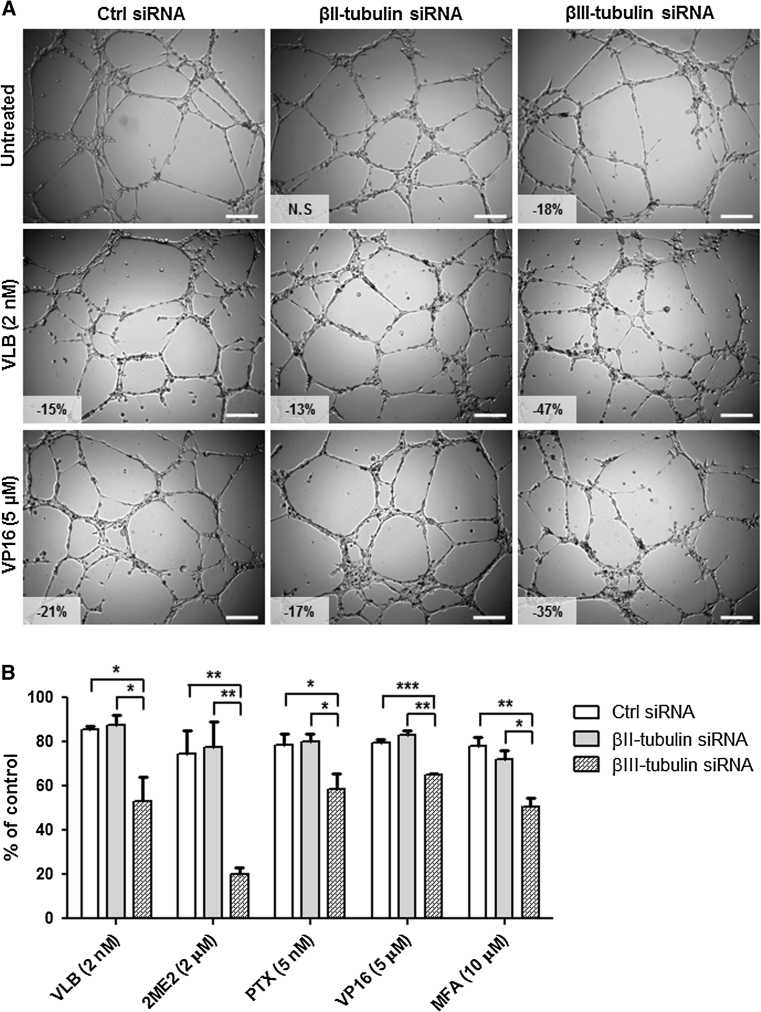
Impact of βII- and βIII-tubulin knockdown on the sensitivity of endothelial cells to the anti-angiogenic effects of chemotherapy. **a** Representative photographs of siRNA-transfected HMEC-1 cells incubated for 8 h on Matrigel in the absence of drug (*top panel*) or in presence of vinblastine at 2 nM (*middle panel*) and etoposide at 5 μM (*bottom panel*). Vascular structures were imaged on a Zeiss Axiovert 200 M using a 5X objective. Percentage of angiogenesis inhibition as compared to untreated control siRNA-transfected cells is indicated; *NS* non-significant, *scale bar* 250 μm. **b** Percentage of vascular structure formation by siRNA-transfected HMEC-1 cells following 8 h incubation on Matrigel with 2 nM vinblastine (*VLB*), 2 μM 2-methoxyestradiol (*2ME2*), 5 nM paclitaxel (*PTX*), 5 μM etoposide (*VP16*) and 10 μM mafosfamide (*MFA*), as compared to control untreated cells. *Columns* means of at least three individual experiments, *bars* SE. Statistics were calculated by comparing the mean surface occupied by closed vascular structures per view field (at least 10 view fields per condition); *p < 0.05; **p < 0.01; ***p < 0.001

To evaluate the implication of βII- and βIII-tubulin expression in the modulation of the angiogenic potential and chemosensitivity of endothelial cells following repeated exposure to chemotherapy, functional analysis using small interfering RNA (siRNA) was undertaken. HMEC-1 microvascular endothelial cells were transfected with siRNA targeting βII- and βIII-tubulin expression. After 72 h incubation, this resulted in a 92 and 77 % decrease in βII- and βIII-tubulin protein expression, respectively, without any significant compensatory effect on the expression of other tubulin isotypes (Fig. 5a; *p* < 0.001). Knocking-down βII- or βIII-tubulin expression had no effect on the proliferation rate of endothelial cells or the organisation of the tubulin cytoskeleton (data not shown). However, silencing βIII-tubulin expression, but not βII-tubulin, decreased by 18 % the capacity of HMEC-1 cells to form capillary-like tubes on Matrigel™ in vitro (Figs. 5b, 6a, *top panel*; *p* < 0.001). This suggests that βIII-tubulin is, at least in part, involved in the morphological differentiation of endothelial cells into vascular structures.

**Fig. 7:**
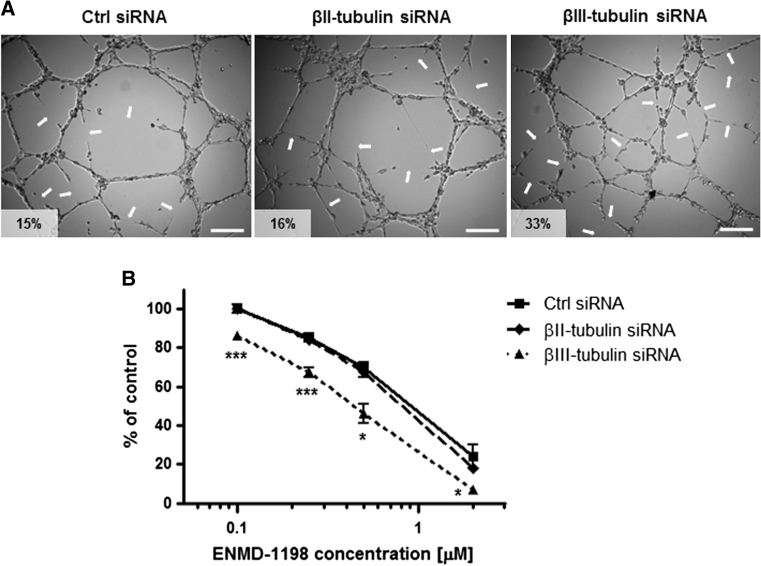
Impact of βII- and βIII-tubulin knockdown on the sensitivity of endothelial cells to vascular-disrupting agent ENMD-1198. **a** Representative photographs of siRNA-transfected HMEC-1 cells in vascular-disruption assay. Cells were first allowed to form vascular structures on Matrigel for 6 h before drug treatment was initiated. Cells were then incubated for 2 h in presence of ENMD-1198 at 0.25 μM and vascular structures were imaged on a Zeiss Axiovert 200 M using a 5X objective. *Arrows* point to collapsing and regressing vascular structures. Percentage of vascular disruption is indicated; *Scale bar* 250 μm. **b** Dose-dependent effect of ENMD-1198 on the disruption of capillary-like structures formed by siRNA-transfected HMEC-1 cells, after 2 h drug incubation. *Points* % of intact vascular structures as compared to untreated control cells, means of at least three individual experiments, *bars* SE; log scale for x axis. Statistics were calculated by comparing the mean surface occupied by closed vascular structures per view field (at least 10 view fields per condition); *p < 0.05; ***p < 0.001

Short-term growth inhibition assay then showed that silencing βII- or βIII-tubulin expression only marginally increased, if at all, the sensitivity of endothelial cells to the anti-proliferative effects of chemotherapy agents. This lack of sensitization was observed with vinblastine (Fig. 5c), etoposide (Fig. 5d), paclitaxel (Fig. 5d), 2-methoxyestradiol (2ME2, Fig. 5e), and other chemotherapeutic drugs such as 2ME2 analogue ENMD-1198 and mafosfamide (MFA)—an analogue of cyclophosphamide active in vitro—(data not shown). In contrast with the lack of effect on proliferation inhibition, silencing βIII-tubulin expression, but not βII-tubulin, significantly increased the sensitivity of endothelial cells to the anti-angiogenic effects of chemotherapy. Incubation with 2 nM vinblastine inhibited vessel formation by 15 and 13 % in control siRNA- and βII-tubulin siRNA-treated HMEC-1 cells, respectively, while the same drug treatment resulted in a 47 % angiogenesis inhibition in βIII-tubulin siRNA-treated cells (Fig. 6a, *middle panel*; *p* < 0.05). Similarly, incubation with 5 μM etoposide inhibited vessel formation by 35 % in βIII-tubulin siRNA-treated HMEC-1 cells, as compared to 21 and 17 % in control siRNA- and βII-tubulin siRNA-treated cells, respectively (Fig. 6a, *bottom panel*; *p* < 0.01). The sensitization of endothelial cells to the anti-angiogenic effects of chemotherapy following βIII-tubulin knockdown was further confirmed with other chemotherapy agents, such as 2-methoxyestradiol, paclitaxel and mafosfamide (Fig. 6b), and using BMH29L cells transfected with siRNA (Online Resource 3). Finally, βIII-tubulin knockdown was also found to sensitize endothelial cells to the vascular-disrupting activity of 2-methoxyestradiol analogue ENMD-1198 (Fig. 7). Incubation with ENMD-1198 at 0.25 μM for 2 h resulted in 5–8 disruption marks per view field in the vascular network formed by control and βII-tubulin siRNA-treated HMEC-1 cells on Matrigel, whereas the same drug treatment resulted in 10–15 disruption marks in the vascular network formed by βIII-tubulin siRNA-treated cells (Fig. 7a; *p* < 0.001). Quantitative analysis of vascular disruption showed that a significant difference was observed across a range of ENMD-1198 concentrations (0.1–2 μM; Fig. 7b). Collectively, our results demonstrate that βIII-tubulin expression is a determining factor in the anti-vascular activity of chemotherapy agents.

## Discussion

Inhibition of tumour angiogenesis is a recognized mechanism of action of chemotherapy [2, 3, 34]. However, the long-term effects of chemotherapeutic drugs on vascular endothelial cells have not been explored. In this study, we used bone-marrow derived endothelial cells immortalized by ectopic expression of human telomerase reverse transcriptase [21] to analyse the impact of repeated exposure to chemotherapy on the angiogenic potential and chemosensitivity of vascular endothelial cells. We found that the long-term effects of chemotherapy on endothelial cells were drug-, concentration- and schedule-dependent.

First, we demonstrated that repeated exposure to vinblastine at the maximum-tolerated dose (MTD) increased the angiogenic potential of vascular endothelial cells by promoting their proliferation without altering their capacity to form vascular structures on Matrigel. Furthermore, this treatment regimen led to a 1.8-fold increase in paclitaxel resistance in endothelial cells. Recently, VEGF was found to increase resistance to paclitaxel in tumour endothelial cells by inducing *ABCB1* expression [18]. Previous studies have also reported that endothelial cells isolated from tumour tissues, such as hepatocellular carcinoma [16] and malignant glioma [17], displayed increased angiogenic potential and intrinsic drug resistance, which may limit the effectiveness of chemotherapy. Here, we provide the first evidence that endothelial cells can acquire some level of drug resistance as a result of repeated exposure to chemotherapy in vitro and may contribute to drug resistance acquisition in tumours in vivo following MTD chemotherapy. Further investigations are currently underway to validate our findings in vivo.

The increase in angiogenic potential and drug resistance induced by MTD chemotherapy was in marked contrast with the long-term effects of low-dose metronomic (LDM) chemotherapy. Indeed, our results demonstrated that repeated exposure to LDM chemotherapy led to sustained impairment of the angiogenic potential of endothelial cells and significantly increased their chemosensitivity. Cells treated with LDM chemotherapy thus exhibited a slower proliferation rate, decreased capacity to form vascular structures on Matrigel and increased sensitivity to the anti-proliferative and anti-angiogenic effects of chemotherapy agents.

Our findings are in accordance with previous studies showing that the presence and length of treatment-free intervals are major determinants in the development of drug resistance in tumour cells, and that drug resistance acquisition can be hindered, prevented and even circumvented simply by modifying the dose and schedule of chemotherapy administration [1, 19, 20]. In the seminal study that led to the development of metronomic chemotherapy, Browder et al. demonstrated that using more frequent cyclophosphamide administration could re-sensitize Lewis lung carcinoma tumours that were made resistant to the same drug [1]. More recently, cyclophosphamide was found to generate dramatically different resistance phenotypes in prostate and breast cancer cells in vivo, depending on the dose and schedule of treatment [20]. While cancer cells made resistant to MTD cyclophosphamide in vivo showed stable drug resistance in vitro and in vivo, cells made resistant to LDM cyclophosphamide in vivo retained sensitivity to chemotherapy agents in vitro and to MTD cyclophosphamide in vivo. Here, we report differential effects of vinblastine in endothelial cells when administered at the MTD or following a LDM schedule, similar to that observed with taxanes and cyclophosphamide in tumour cells. In contrast, the effects of etoposide appeared to be less schedule-dependent in endothelial cells and repeated exposure led to impaired angiogenic potential and increased chemosensitivity, irrespective of the treatment schedule.

Since intracellular drug accumulation is the basic requirement for chemotherapy agents to exert their effects, upregulation of ABC transporters is of crucial importance in drug resistance acquisition [35]. Here we found that repeated exposure to chemotherapy induced a significant upregulation of *ABCB1* gene expression, irrespective of the treatment schedule and the type of chemotherapy agent used. In contrast, the effects on *ABCC2* gene expression were schedule-dependent, with an increase observed in *VLB*-*MTD* cells and a decrease in *VLB*-*LDM* and *VP16*-*LDM* cells. However, the changes in ABC transporter gene expression did not correlate with major changes in intracellular drug accumulation. All 6 endothelial subclones were found to accumulate very high levels of tritiated vincristine (~45 pmol per mg of protein), which was two- to six-fold higher than in neuroblastoma cells. This high vincristine uptake was unaltered by co-administration of verapamil, suggesting that the increase in *ABCB1* gene expression did not translate into increased drug efflux. Interestingly, increased uptake of paclitaxel in endothelial cells was previously reported and proposed as a potential mechanism for the anti-angiogenic activity of paclitaxel [36]. Here we demonstrated that endothelial uptake of *Vinca* alkaloids is also high and may contribute to their potent anti-vascular properties [37].

The lack of correlation between changes in ABC transporter expression and intracellular drug accumulation strongly suggests that the effects of repeated exposure to chemotherapy on the drug sensitivity of endothelial cells are not mediated by ABC transporters. We therefore sought to investigate other candidate genes that may be involved. The expression level of specific β-tubulin isotypes has been shown to influence drug sensitivity in a range of cancer cell lines and malignancies, including lung, ovarian, breast and prostate cancers (reviewed in [33]). Here we found that changes in chemosensitivity of endothelial cells induced by repeated exposure to chemotherapy were associated with significant changes in β-tubulin isotype expression. In particular, all 3 BMH29L subclones displaying increased drug sensitivity (*VLB*-*LDM*, *VP16*-*LDM* and *VP16*-*MTD*) were found to express lower protein levels of βII- and βIII-tubulin. This is the first demonstration that repeated exposure to microtubule-targeting agents, such as vinblastine, and other classes of chemotherapy agent, such as the topoisomerase inhibitor etoposide, can result in significant changes in tubulin isotype expression in non-cancer cells, most likely through transcriptional repression.

Using a series of elegant experiments, Piquette-Miller and colleagues demonstrated that continuous administration of taxanes (*i.e.* paclitaxel and docetaxel) was considerably more efficacious than intermittent taxane therapy (i.e. once or thrice weekly) against ovarian cancer cells in vitro and in vivo [19, 38, 39]. Interestingly, intermittent docetaxel treatment of ovarian tumours led to significant upregulation of *ABCB1*, *ABCC10*, *bcl2* and *TUBB3* among other genes involved in drug resistance whereas continuous docetaxel did not induce upregulation of any analysed genes but instead led to a downregulation of *ABCC10*, *TUBB3* and *stathmin* [19]. Our results are in accordance with these studies and confirm the influence of treatment schedule on the modulation of the expression of genes involved in drug resistance not only in tumour cells but also in endothelial cells.

We previously reported relatively high expression of βII- and βIII-tubulin in vascular endothelial cells [40] and showed that both these tubulin isotypes strongly influence the response of non-small cell lung carcinoma cells (NSCLC) to chemotherapeutic drugs [25, 28, 29, 41]. In the present study, functional analysis using gene silencing technology showed that βIII-tubulin, but not βII-tubulin, plays a crucial role in the anti-angiogenic activity of chemotherapy. Knocking down βIII-tubulin expression thus resulted in a small, but significant, decrease in the angiogenic potential of endothelial cells and increased their sensitivity to the anti-angiogenic and vascular-disrupting activity of chemotherapeutic drugs. This finding provides further evidence of the key role played by βIII-tubulin in tumour progression, angiogenesis and drug resistance. Recently, βIII-tubulin was identified as a marker of angiogenic perivascular cells in rat mesenteric tissues during active capillary sprouting [42]. Furthermore, a large number of studies have linked abnormal or high levels of βIII-tubulin expression with more aggressive and drug-resistant phenotypes in a range of tumour types, including lung, ovarian, breast and gastric cancers (reviewed in [33, 43]). In NSCLC for instance, expression of βIII-tubulin is associated with poorly differentiated tumour tissue, high-grade malignancy and increased metastatic potential [43], as well as lower response rate to paclitaxel/vinorelbine-based chemotherapy and shorter progression-free and overall survival [44, 45]. Collectively the results of our current study together with our previous work [25, 28, 29, 41] and accumulating clinical data strongly suggest that βIII-tubulin represents an attractive therapeutic target to increase the anti-angiogenic effects of chemotherapy and overall anti-tumour efficacy.

## Electronic supplementary material

Below is the link to the electronic supplementary material.

**Figure 1. Long-term treatment of vascular endothelial cells with chemotherapy.** (**A**) Growth inhibition assay performed on BMH29L cells using Alamar Blue after 72 h incubation with a range of concentrations of vinblastine (*red*) and etoposide (*green*). *Points*, % of cell proliferation as compared to untreated control cells, means of at least three individual experiments; *bars*, SE; log scale for x axis; vertical lines indicate the highest non-toxic drug concentration and the IC_80_. (**B**) Table showing the highest non-toxic drug concentration and IC_80_ for vinblastine and etoposide that were subsequently used for long-term drug treatment. (**C**) Schematic representing the two treatment schedules over 100 days. For the low-dose metronomic (LDM) chemotherapy, BMH29L cells were treated 5 days a week with the highest non-toxic concentration of either vinblastine or etoposide for a total of 14 cycles. For the maximum-tolerated dose (MTD) chemotherapy, BMH29L were treated once every 2 weeks with the IC_80_ of either vinblastine or etoposide for a total of 7 cycles. (PDF 194 kb)

**Figure 2. Impact of repeated exposure to chemotherapy on β-tubulin gene expression.** Histogram showing the relative expression of β-tubulin genes *TUBB*, *TUBB2A* and *TUBB3* (encoding for βI-, βII- and βIII-tubulin, respectively) in the 6 BMH29L subclones as determined by quantitative RT-PCR after normalization to the *GADPH* control. *Columns,* means of three individual experiments; *bars*, SE. Statistics were calculated by comparing drug-treated cells with control cells; * p < 0.05, ** p < 0.01. (PDF 193 kb)

**Figure 3. Confirmation of the impact of βIII-tubulin knockdown on the sensitivity of endothelial cells to the anti-angiogenic effects of chemotherapy.** (**A**) Representative immunoblots of whole cell lysates, 72 h after transfection of BMH29L cells with negative control, βII- and βIII-tubulin siRNA. Membranes were probed with antibodies directed against βII-, βIII- and total β-tubulin. (**B**) Mean surface occupied by vascular structures formed by BMH29L cells 72 h after siRNA transfection and following 8 h incubation on Matrigel. *Boxes*, min–max range of 4 individual experiments; *bars*, SD; * p < 0.05. (**C**) Representative photographs of siRNA-transfected BMH29L cells incubated for 8 h on Matrigel in the absence of drug (*top panel*) or in presence of vinblastine at 10 nM (*middle panel*) and etoposide at 50 µM (*bottom panel*). Vascular structures were imaged on a Zeiss Axiovert 200 M using a 5 X objective. Percentage of angiogenesis inhibition as compared to untreated control siRNA-transfected cells is indicated; *N.S*, non-significant; *Scale bar*, 250 µm. (PDF 1222 kb)

## Abbreviation

2ME2: 2-Methoxyestradiol
[^3^H]-VCR: Tritiated vincristine
ABC: ATP-binding cassette
BMECs: Bone marrow derived endothelial cells
FCS: Fetal calf serum
FGF_β_: Fibroblast growth factor β
GADPH: Glyceraldehyde-3-phosphate dehydrogenase
HMEC-1: Human microvascular endothelial cell line 1
LDM: Low-dose metronomic
MFA: Mafosfamide
MTD: Maximum tolerated dose
PTX: Paclitaxel
RT-PCR: Reverse-transcriptase polymerase chain reaction
siRNA: Small interfering RNA
VLB: Vinblastine
VP16: Etoposide
