# Supplementary material for: Concentration- and schedule-dependent effects of chemotherapy on the angiogenic potential and drug sensitivity of vascular endothelial cells
Source: Angiogenesis. 2012 Nov 10;16(2):373–86. doi: 10.1007/s10456-012-9321-x (PMC3595478; doi:10.1007/s10456-012-9321-x)

Title: Concentration- and schedule-dependent effects of chemotherapy on the angiogenic potential and drug sensitivity of vascular endothelial cells

Journal: Angiogenesis

Authors: Eddy Pasquier[1,2], Maria-Pia Tuset[1], Janine Street[1], Snega Sinnappan[1], Karen MacKenzie[1], Diane Braguer[3], Nicolas Andre[2,3,4] and Maria Kavallaris[1,5]

Affiliations: 1) Children's Cancer Institute Australia, Lowy Cancer Research Centre, UNSW, Randwick, NSW, Australia
2) Metronomics Global Health Initiative, Marseille, France
3) INSERM UMR 911, Centre de Recherche en Oncologie biologique et en Oncopharmacologie, Aix-Marseille University, Faculty of Pharmacy, Marseille, France
4) Hematology and Pediatric Oncology Department, La Timone University Hospital of Marseille, France
5) Australian Centre for Nanomedicine, University of New South Wales, NSW, 2051, Australia

Corresponding author: Maria Kavallaris PhD
E-mail: m.kavallaris@ccia.unsw.edu.au

A

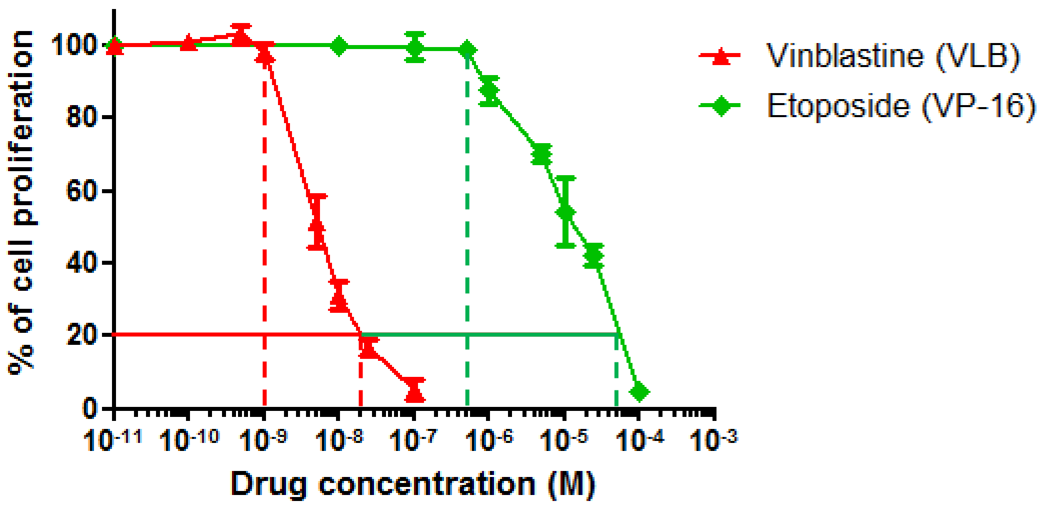

B

Type of treatment	LDM (IC_1)	MTD (IC_{80})
Vinblastine	1 nM	20 nM
Etoposide	0.5 µM	50 µM

C

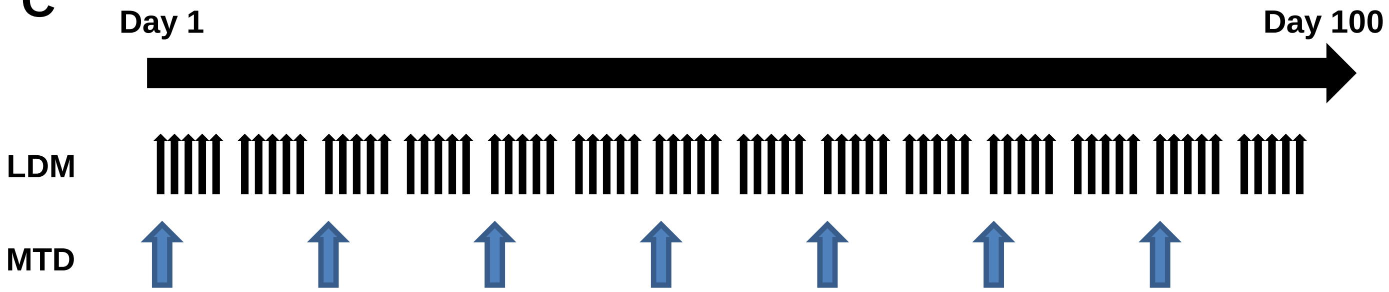

Supplement: Supplementary file 1 — Figure 1. Long-term treatment of vascular endothelial cells with chemotherapy. (A) Growth inhibition assay performed on BMH29L cells using Alamar Blue after 72 h incubation with a range of concentrations of vinblastine (red) and etoposide (green). Points, % of cell proliferation as compared to untreated control cells, means of at least three individual experiments; bars, SE; log scale for x axis; vertical lines indicate the highest non-toxic drug concentration and the IC80. (B) Table showing the highest non-toxic drug concentration and IC80 for vinblastine and etoposide that were subsequently used for long-term drug treatment. (C) Schematic representing the two treatment schedules over 100 days. For the low-dose metronomic (LDM) chemotherapy, BMH29L cells were treated 5 days a week with the highest non-toxic concentration of either vinblastine or etoposide for a total of 14 cycles. For the maximum-tolerated dose (MTD) chemotherapy, BMH29L were treated once every 2 weeks with the IC80 of either vinblastine or etoposide for a total of 7 cycles. (PDF 194 kb) [file 10456_2012_9321_MOESM1_ESM.pdf]
