# Supplementary material for: Concentration- and schedule-dependent effects of chemotherapy on the angiogenic potential and drug sensitivity of vascular endothelial cells
Source: Angiogenesis. 2012 Nov 10;16(2):373–86. doi: 10.1007/s10456-012-9321-x (PMC3595478; doi:10.1007/s10456-012-9321-x)
Supplement: Supplementary file 2 — Figure 2. Impact of repeated exposure to chemotherapy on β-tubulin gene expression. Histogram showing the relative expression of β-tubulin genes TUBB, TUBB2A and TUBB3 (encoding for βI-, βII- and βIII-tubulin, respectively) in the 6 BMH29L subclones as determined by quantitative RT-PCR after normalization to the GADPH control. Columns, means of three individual experiments; bars, SE. Statistics were calculated by comparing drug-treated cells with control cells; * p < 0.05, ** p < 0.01. (PDF 193 kb) [file 10456_2012_9321_MOESM2_ESM.pdf]

Title: Concentration- and schedule-dependent effects of chemotherapy on the angiogenic potential and drug sensitivity of vascular endothelial cells

Journal: Angiogenesis

Authors: Eddy Pasquier[1,2], Maria-Pia Tuset[1], Janine Street[1], Snega Sinnappan[1], Karen MacKenzie[1], Diane Braguer[3], Nicolas Andre[2,3,4] and Maria Kavallaris[1,5]

Affiliations: 1) Children's Cancer Institute Australia, Lowy Cancer Research Centre, UNSW, Randwick, NSW, Australia
2) Metronomics Global Health Initiative, Marseille, France
3) INSERM UMR 911, Centre de Recherche en Oncologie biologique et en Oncopharmacologie, Aix-Marseille University, Faculty of Pharmacy, Marseille, France
4) Hematology and Pediatric Oncology Department, La Timone University Hospital of Marseille, France
5) Australian Centre for Nanomedicine, University of New South Wales, NSW, 2051, Australia

Corresponding author: Maria Kavallaris PhD
E-mail: m.kavallaris@ccia.unsw.edu.au

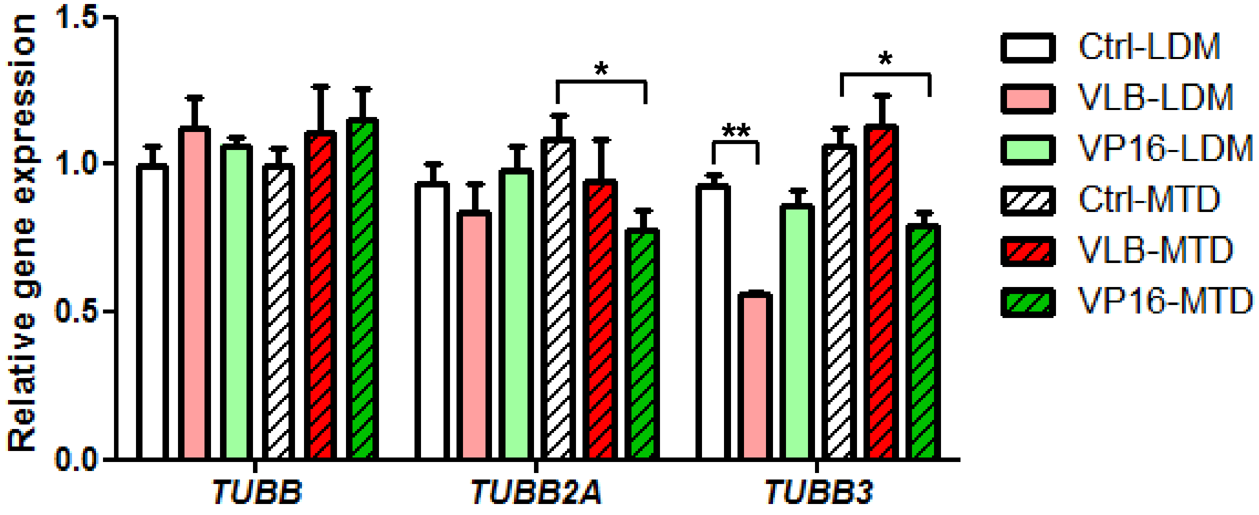
1.5
1.0
0.5
0.0
Relative gene expression
TUBB
TUBB2A
TUBB3
*
**
*
Ctrl-LDM
VLB-LDM
VP16-LDM
Ctrl-MTD
VLB-MTD
VP16-MTD
