# Supplementary material for: Concentration- and schedule-dependent effects of chemotherapy on the angiogenic potential and drug sensitivity of vascular endothelial cells
Source: Angiogenesis. 2012 Nov 10;16(2):373–86. doi: 10.1007/s10456-012-9321-x (PMC3595478; doi:10.1007/s10456-012-9321-x)
Supplement: Supplementary file 3 — Figure 3. Confirmation of the impact of βIII-tubulin knockdown on the sensitivity of endothelial cells to the anti-angiogenic effects of chemotherapy. (A) Representative immunoblots of whole cell lysates, 72 h after transfection of BMH29L cells with negative control, βII- and βIII-tubulin siRNA. Membranes were probed with antibodies directed against βII-, βIII- and total β-tubulin. (B) Mean surface occupied by vascular structures formed by BMH29L cells 72 h after siRNA transfection and following 8 h incubation on Matrigel. Boxes, min–max range of 4 individual experiments; bars, SD; * p < 0.05. (C) Representative photographs of siRNA-transfected BMH29L cells incubated for 8 h on Matrigel in the absence of drug (top panel) or in presence of vinblastine at 10 nM (middle panel) and etoposide at 50 µM (bottom panel). Vascular structures were imaged on a Zeiss Axiovert 200 M using a 5 X objective. Percentage of angiogenesis inhibition as compared to untreated control siRNA-transfected cells is indicated; N.S, non-significant; Scale bar, 250 µm. (PDF 1222 kb) [file 10456_2012_9321_MOESM3_ESM.pdf]

Title: Concentration- and schedule-dependent effects of chemotherapy on the angiogenic potential and drug sensitivity of vascular endothelial cells

Journal: Angiogenesis

Authors: Eddy Pasquier[1,2], Maria-Pia Tuset[1], Janine Street[1], Snega Sinnappan[1], Karen MacKenzie[1], Diane Braguer[3], Nicolas Andre[2,3,4] and Maria Kavallaris[1,5]

Affiliations: 1) Children's Cancer Institute Australia, Lowy Cancer Research Centre, UNSW, Randwick, NSW, Australia
2) Metronomics Global Health Initiative, Marseille, France
3) INSERM UMR 911, Centre de Recherche en Oncologie biologique et en Oncopharmacologie, Aix-Marseille University, Faculty of Pharmacy, Marseille, France
4) Hematology and Pediatric Oncology Department, La Timone University Hospital of Marseille, France
5) Australian Centre for Nanomedicine, University of New South Wales, NSW, 2051, Australia

Corresponding author: Maria Kavallaris PhD
E-mail: m.kavallaris@ccia.unsw.edu.au

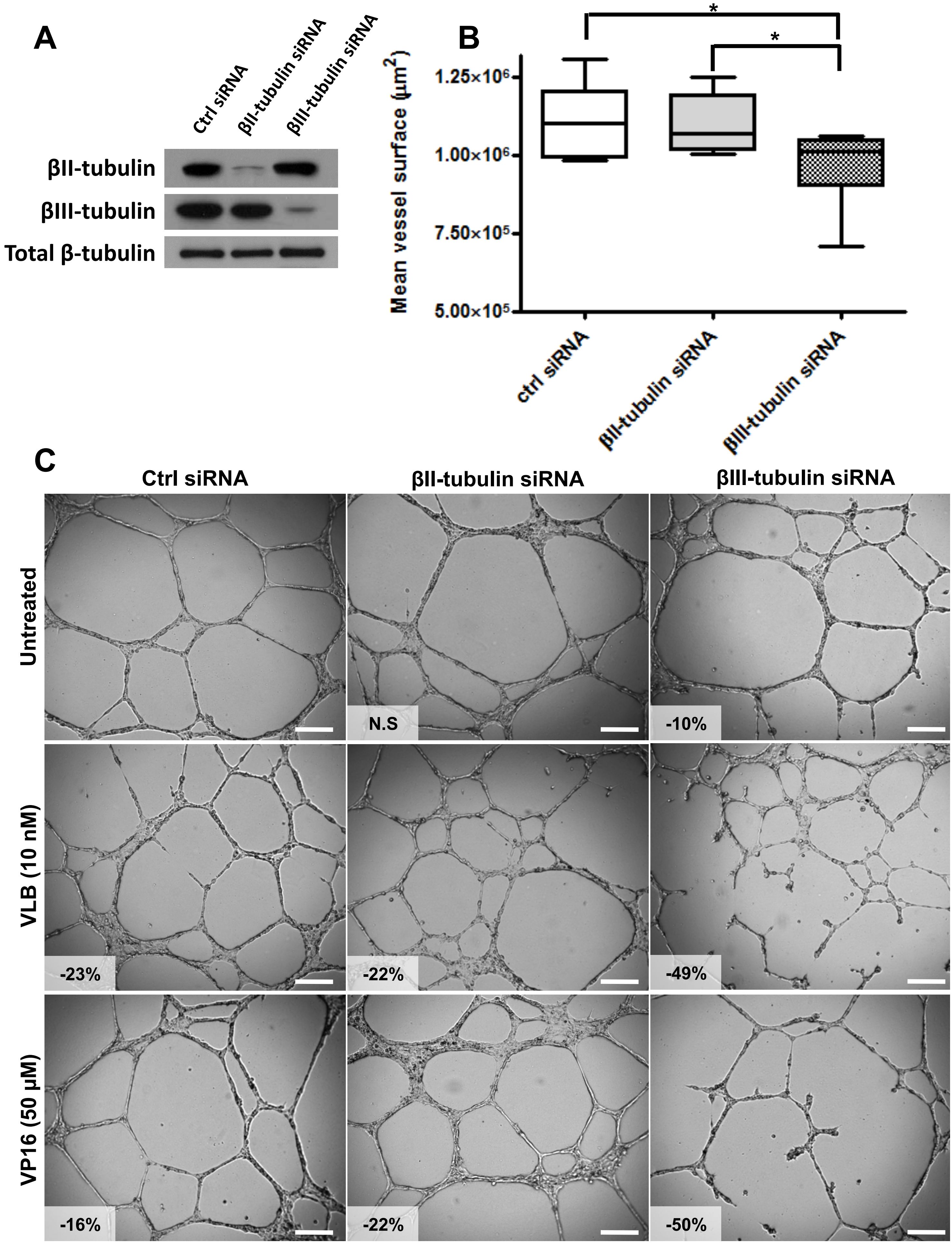
A
Ctrl siRNA
βII-tubulin siRNA
βIII-tubulin siRNA
βII-tubulin
βIII-tubulin
Total β-tubulin
B
Mean vessel surface (μm2)
1.25×10⁶
1.00×10⁶
7.50×10⁵
5.00×10⁵
*
*
ctrl siRNA
βII-tubulin siRNA
βIII-tubulin siRNA
C
Ctrl siRNA
βII-tubulin siRNA
βIII-tubulin siRNA
Untreated
VLB (10 nM)
VP16 (50 μM)
N.S
-10%
-23%
-22%
-49%
-16%
-22%
-50%
